# A new polymorph of Lu(PO_3_)_3_

**DOI:** 10.1107/S1600536808021995

**Published:** 2008-07-19

**Authors:** Anis Bejaoui, Karima Horchani-Naifer, Mokhtar Férid

**Affiliations:** a Unité de Recherches de Matériaux de Terres Rares, Centre National de Recherches en Sciences des Matériaux, BP 95 Hammam-Lif 2050, Tunisia

## Abstract

A new polymorph of lutetium polyphosphate, Lu(PO_3_)_3_, was found to be isotypic with the trigonal form of Yb(PO_3_)_3_. Two of the three Lu atoms occupy special positions (Wyckoff positions 3*a* and 3*b*, site symmetry 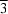). The atomic arrangement consists of infinite helical polyphosphate chains running along the *c* axis, with a repeat period of 12 PO_4_ tetra­hedra, joined with LuO_6_ octa­hedra.

## Related literature

For syntheses and optical properties, see: Briche *et al.* (2006 ▶); Jouini, Férid, Gacon, Grosvalet *et al.* (2003 ▶); Jouini, Férid, Gacon & Trabelsi-Ayadi (2003 ▶); Ternane *et al.* (2005 ▶); Graia *et al.* (2003 ▶); Anisimova *et al.* (1992 ▶). For the monoclinic polymorph of Lu(PO_3_)_3_, see: Höppe & Sedlmaier (2007 ▶); Yuan *et al.* (2008 ▶).

## Experimental

### 

#### Crystal data

Lu(PO_3_)_3_
*M* *_r_* = 411.88
Trigonal, 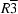

*a* = 20.9106 (6) Å
*c* = 12.0859 (7) Å
*V* = 4576.6 (3) Å^3^
*Z* = 24
Mo *K*α radiation
μ = 13.59 mm^−1^
*T* = 100 (2) K
0.18 × 0.18 × 0.17 mm

#### Data collection

Bruker APEXII CCD area-detector diffractometer
Absorption correction: multi-scan (*SADABS*; Sheldrick, 1996 ▶) *T*_min_ = 0.102, *T*_max_ = 0.104
25139 measured reflections
4170 independent reflections
3609 reflections with *I* > 2σ(*I*)
*R*_int_ = 0.054

#### Refinement

*R*[*F* ^2^ > 2σ(*F* ^2^)] = 0.032
*wR*(*F* ^2^) = 0.060
*S* = 1.05
4170 reflections
159 parameters
Δρ_max_ = 2.34 e Å^−3^
Δρ_min_ = −2.07 e Å^−3^

### 

Data collection: *APEX2* (Bruker, 2005 ▶); cell refinement: *APEX2*; data reduction: *APEX2*; program(s) used to solve structure: *SHELXS97* (Sheldrick, 2008 ▶); program(s) used to refine structure: *SHELXL97* (Sheldrick, 2008 ▶); molecular graphics: *DIAMOND* (Brandenburg, 2001 ▶); software used to prepare material for publication: *SHELXL97*.

## Supplementary Material

Crystal structure: contains datablocks I, global. DOI: 10.1107/S1600536808021995/fi2065sup1.cif

Structure factors: contains datablocks I. DOI: 10.1107/S1600536808021995/fi2065Isup2.hkl

Additional supplementary materials:  crystallographic information; 3D view; checkCIF report

## Figures and Tables

**Fig. 1.**
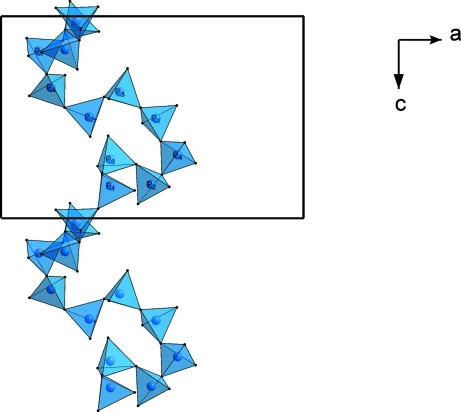
A projection of the helical (PO3)n chains along b.

**Fig. 2.**
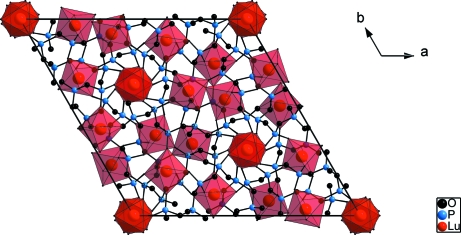
A projection of Lu(PO3)3 along c, showing the arrangement of the LuO6 octahedra and PO4 tetrahedra.

**Fig. 3.**
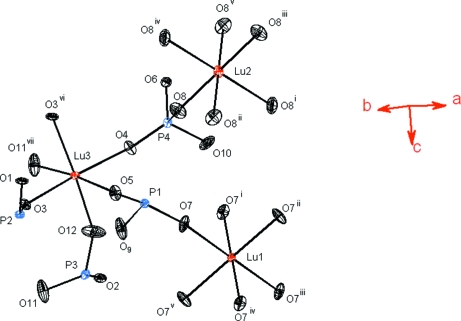
Projection of the Lu(PO3)3 polyphosphate, showing the lutetium coordination with displacement ellipsoids drawn at the 50% probability level. [Symmetry codes : (i) -y, x-y, z; (ii) -x+y, -x, z; (iii)-x+1/3, -y+2/3, -z+2/3; (iv) y+1/3, -x+y+2/3, -z+2/3; (v) x-y+1/3, x+2/3, -z+2/3; (vi) -x, -y, -z; (vii) -x+y+1/3, -x+2/3, z+2/3.]

## supplementary crystallographic information

### Comment

There is considerable scientific and technological interest in the synthesis, structure, and properties of yttrium and rare earth polyphosphates of the formula Ln(PO_3_)_3_, because these compounds offer thermal stability and richness of formulations and structures (Briche *et al.*, 2006, Jouini, Férid, Gacon, Grosvalet *et al.*, 2003, Jouini, Férid, Gacon & Trabelsi-Ayadi, 2003, Ternane *et al.*, 2005, Graia *et al.*, 2003). In this paper, we report the preparation and crystal structure refinement of the polyphosphate Lu(PO_3_)_3_, crystallizing in space group *R*-3 . The existence of the trigonal polymorph was originally reported by Anisimova for the Yb(PO_3_)_3_ polyphosphate (Anisimova *et al.*, 1992). The monoclinic polymorph of Lu(PO_3_)_3_ was recently reported by Höppe and Yuan (Höppe & Sedlmaier, 2007, Yuan *et al.*, 2008). The atomic arrangement of these structures is characterized by a three-dimensional framework built of (PO_3_)_n_ chains that are formed by corner-sharing of PO_4_ tetrahedra. These two polymorphs differ by the polyphosphate chains configuration. The chains that were observed in monoclinic Lu(PO_3_)_3_ form infinite zigzag chains (PO_3_)_n _that extend along *c* with a period of six tetrahedra. In trigonal Lu(PO_3_)_3_, the (PO_3_)_n_ chains are helical with a period of 12 tetrahedra (Fig.1) and are arranged about the 3_1_ helical axis. The chains are joined to each other by LuO_6_ octahedra (Fig 2.), no oxygen atom is shared between adjacent LuO_6_ octahedra. Figure 3 shows the projection of Lu(PO_3_)_3 _with anisotropic displacement parameters drawn at the 50% probability level.

### Experimental

Single crystals of Lu(PO_3_)_3_ were grown by a flux method. Lutetium oxide was dissolved in an excess of phosphoric acid using the molar ratio Lu:*P* = 1:20. The resulting solution was heated in a vitreous graphite crucible at 573 K for 5 days. The obtained colourless crystals were then isolated from the acid solution using hot water.

### Refinement

The highest peak and the deepest hole are located 0.75Å and 0.57 Å, respectively from O10 and Lu3.

### Crystal data

**Table:**

Lu(PO_3_)_3_	*Z* = 24
*M**_r_* = 411.88	*F*_000_ = 4512
Trigonal, *R*3	*D*_x_ = 3.587 Mg m^−^^3^
Hall symbol: -R 3	Mo *K*α radiation λ = 0.71073 Å
*a* = 20.9106 (6) Å	Cell parameters from 25 reflections
*b* = 20.9106 (6) Å	θ = 2.8–34.1º
*c* = 12.0859 (7) Å	µ = 13.59 mm^−^^1^
α = 90º	*T* = 100 (2) K
β = 90º	Cube, colourless
γ = 120º	0.18 × 0.18 × 0.17 mm
*V* = 4576.6 (3) Å^3^	

### Data collection

**Table:**

Bruker APEXII CCD area-detector diffractometer	3609 reflections with *I* > 2σ(*I*)
Monochromator: graphite	*R*_int_ = 0.054
*T* = 100(2) K	θ_max_ = 34.2º
ω scans	θ_min_ = 2.0º
Absorption correction: multi-scan(SADABS; Sheldrick, 1996)	*h* = −32→32
*T*_min_ = 0.102, *T*_max_ = 0.104	*k* = −32→32
25139 measured reflections	*l* = −18→18
4170 independent reflections	

### Refinement

**Table:**

Refinement on *F*^2^	Secondary atom site location: difference Fourier map
Least-squares matrix: full	*w* = 1/[σ^2^(*F*_o_^2^) + (0.0212*P*)^2^] where *P* = (*F*_o_^2^ + 2*F*_c_^2^)/3
*R*[*F*^2^ > 2σ(*F*^2^)] = 0.032	(Δ/σ)_max_ = 0.001
*wR*(*F*^2^) = 0.061	Δρ_max_ = 2.34 e Å^−^^3^
*S* = 1.05	Δρ_min_ = −2.07 e Å^−^^3^
4170 reflections	Extinction correction: SHELXL97 (Sheldrick, 2008), Fc^*^=kFc[1+0.001xFc^2^λ^3^/sin(2θ)]^-1/4^
159 parameters	Extinction coefficient: 0.000061 (8)
Primary atom site location: structure-invariant direct methods	

### Special details

**Table:**

Geometry. All e.s.d.'s (except the e.s.d. in the dihedral angle between two l.s. planes) are estimated using the full covariance matrix. The cell e.s.d.'s are taken into account individually in the estimation of e.s.d.'s in distances, angles and torsion angles; correlations between e.s.d.'s in cell parameters are only used when they are defined by crystal symmetry. An approximate (isotropic) treatment of cell e.s.d.'s is used for estimating e.s.d.'s involving l.s. planes.
Refinement. Refinement of *F*^2^ against ALL reflections. The weighted *R*-factor *wR* and goodness of fit *S* are based on *F*^2^, conventional *R*-factors *R* are based on *F*, with *F* set to zero for negative *F*^2^. The threshold expression of *F*^2^ > σ(*F*^2^) is used only for calculating *R*-factors(gt) *etc*. and is not relevant to the choice of reflections for refinement. *R*-factors based on *F*^2^ are statistically about twice as large as those based on *F*, and *R*- factors based on ALL data will be even larger.

### Fractional atomic coordinates and isotropic or equivalent isotropic displacement parameters (Å^2^)

**Table:**

	*x*	*y*	*z*	*U*_iso_*/*U*_eq_	
Lu1	0.6667	0.3333	0.3333	0.00741 (8)	
Lu2	0.6667	0.3333	−0.1667	0.01207 (9)	
Lu3	0.440661 (9)	0.365196 (10)	0.096806 (14)	0.00780 (5)	
P1	0.63820 (6)	0.45920 (6)	0.16119 (9)	0.00821 (19)	
P2	0.50313 (6)	0.54494 (6)	0.16810 (9)	0.0096 (2)	
P3	0.39267 (6)	0.30556 (6)	0.37383 (10)	0.0111 (2)	
P4	0.50120 (6)	0.25039 (6)	−0.01904 (10)	0.0107 (2)	
O1	0.44368 (17)	0.46669 (17)	0.1552 (3)	0.0132 (6)	
O2	0.34108 (18)	0.22020 (17)	0.3991 (3)	0.0132 (6)	
O3	0.54706 (18)	0.58558 (18)	0.0709 (3)	0.0141 (6)	
O4	0.45503 (18)	0.27392 (18)	0.0416 (3)	0.0168 (7)	
O5	0.55847 (17)	0.42399 (18)	0.1374 (3)	0.0175 (7)	
O6	0.45659 (17)	0.19780 (19)	−0.1185 (3)	0.0155 (7)	
O7	0.66627 (18)	0.41823 (18)	0.2253 (3)	0.0190 (7)	
O8	0.57293 (19)	0.3097 (2)	−0.0609 (3)	0.0247 (8)	
O9	0.6655 (2)	0.5377 (2)	0.2137 (4)	0.0351 (11)	
O10	0.5156 (2)	0.1948 (2)	0.0471 (3)	0.0315 (10)	
O11	0.3569 (2)	0.3473 (2)	0.4088 (4)	0.0298 (9)	
O12	0.4182 (3)	0.3127 (2)	0.2586 (3)	0.0364 (11)	

### Atomic displacement parameters (Å^2^)

**Table:**

	*U*^11^	*U*^22^	*U*^33^	*U*^12^	*U*^13^	*U*^23^
Lu1	0.00684 (11)	0.00684 (11)	0.00854 (19)	0.00342 (6)	0.000	0.000
Lu2	0.01014 (13)	0.01014 (13)	0.0159 (2)	0.00507 (6)	0.000	0.000
Lu3	0.00810 (8)	0.00796 (8)	0.00662 (8)	0.00347 (7)	0.00035 (6)	0.00000 (6)
P1	0.0086 (5)	0.0070 (5)	0.0084 (5)	0.0034 (4)	−0.0005 (4)	−0.0006 (4)
P2	0.0133 (5)	0.0095 (5)	0.0072 (5)	0.0065 (4)	−0.0008 (4)	−0.0007 (4)
P3	0.0150 (5)	0.0121 (5)	0.0095 (5)	0.0093 (4)	0.0006 (4)	0.0032 (4)
P4	0.0115 (5)	0.0132 (5)	0.0106 (5)	0.0084 (4)	−0.0009 (4)	0.0000 (4)
O1	0.0157 (15)	0.0117 (14)	0.0132 (15)	0.0076 (13)	0.0006 (12)	−0.0027 (12)
O2	0.0192 (16)	0.0124 (15)	0.0079 (14)	0.0077 (13)	0.0020 (12)	0.0008 (11)
O3	0.0216 (17)	0.0164 (16)	0.0055 (14)	0.0104 (14)	0.0015 (12)	−0.0001 (12)
O4	0.0141 (16)	0.0152 (16)	0.0215 (18)	0.0076 (13)	0.0028 (13)	−0.0036 (13)
O5	0.0089 (14)	0.0147 (16)	0.0269 (19)	0.0044 (13)	−0.0040 (13)	−0.0065 (14)
O6	0.0120 (15)	0.0265 (18)	0.0117 (15)	0.0125 (14)	−0.0042 (12)	−0.0051 (13)
O7	0.0154 (16)	0.0188 (17)	0.0264 (19)	0.0110 (14)	0.0048 (14)	0.0145 (14)
O8	0.0126 (16)	0.027 (2)	0.028 (2)	0.0050 (15)	0.0040 (14)	−0.0063 (16)
O9	0.023 (2)	0.030 (2)	0.060 (3)	0.0199 (18)	−0.019 (2)	−0.030 (2)
O10	0.063 (3)	0.025 (2)	0.021 (2)	0.033 (2)	−0.0207 (19)	−0.0072 (16)
O11	0.0202 (19)	0.0148 (17)	0.058 (3)	0.0114 (15)	0.0067 (18)	−0.0006 (18)
O12	0.063 (3)	0.022 (2)	0.0141 (19)	0.014 (2)	0.0127 (19)	0.0068 (15)

### Geometric parameters (Å, °)

**Table:**

Lu1—O7^i^	2.207 (3)	Lu3—O3^x^	2.229 (3)
Lu1—O7^ii^	2.207 (3)	P1—O5	1.475 (3)
Lu1—O7^iii^	2.207 (3)	P1—O7	1.477 (3)
Lu1—O7^iv^	2.207 (3)	P1—O10^iii^	1.569 (4)
Lu1—O7	2.207 (3)	P1—O9	1.578 (4)
Lu1—O7^v^	2.207 (3)	P2—O3	1.472 (3)
Lu2—O8^vi^	2.180 (3)	P2—O1	1.488 (3)
Lu2—O8^iv^	2.180 (3)	P2—O6^xi^	1.585 (3)
Lu2—O8^iii^	2.180 (3)	P2—O2^ii^	1.593 (3)
Lu2—O8	2.180 (3)	P3—O11	1.467 (4)
Lu2—O8^vii^	2.180 (3)	P3—O12	1.472 (4)
Lu2—O8^viii^	2.180 (3)	P3—O9^i^	1.573 (4)
Lu3—O11^ix^	2.134 (3)	P3—O2	1.587 (3)
Lu3—O12	2.176 (4)	P4—O8	1.478 (4)
Lu3—O4	2.180 (3)	P4—O4	1.478 (3)
Lu3—O5	2.189 (3)	P4—O10	1.560 (4)
Lu3—O1	2.207 (3)	P4—O6	1.581 (3)
			
O7^i^—Lu1—O7^ii^	88.57 (14)	O4—Lu3—O5	87.21 (12)
O7^i^—Lu1—O7^iii^	180.0	O11^ix^—Lu3—O1	91.99 (13)
O7^ii^—Lu1—O7^iii^	91.43 (14)	O12—Lu3—O1	95.32 (14)
O7^i^—Lu1—O7^iv^	91.43 (14)	O4—Lu3—O1	171.70 (12)
O7^ii^—Lu1—O7^iv^	180.0	O5—Lu3—O1	84.56 (12)
O7^iii^—Lu1—O7^iv^	88.57 (14)	O11^ix^—Lu3—O3^x^	88.69 (15)
O7^i^—Lu1—O7	91.43 (14)	O12—Lu3—O3^x^	174.96 (15)
O7^ii^—Lu1—O7	91.43 (14)	O4—Lu3—O3^x^	95.25 (12)
O7^iii^—Lu1—O7	88.57 (14)	O5—Lu3—O3^x^	96.14 (13)
O7^iv^—Lu1—O7	88.57 (14)	O1—Lu3—O3^x^	84.56 (12)
O7^i^—Lu1—O7^v^	88.57 (14)	O5—P1—O7	119.4 (2)
O7^ii^—Lu1—O7^v^	88.57 (14)	O5—P1—O10^iii^	106.7 (2)
O7^iii^—Lu1—O7^v^	91.43 (14)	O7—P1—O10^iii^	110.3 (2)
O7^iv^—Lu1—O7^v^	91.43 (14)	O5—P1—O9	109.15 (19)
O7—Lu1—O7^v^	180.0	O7—P1—O9	110.6 (2)
O8^vi^—Lu2—O8^iv^	180.0	O10^iii^—P1—O9	98.7 (3)
O8^vi^—Lu2—O8^iii^	90.92 (15)	O3—P2—O1	119.23 (19)
O8^iv^—Lu2—O8^iii^	89.08 (15)	O3—P2—O6^xi^	105.71 (18)
O8^vi^—Lu2—O8	90.92 (15)	O1—P2—O6^xi^	109.44 (19)
O8^iv^—Lu2—O8	89.08 (15)	O3—P2—O2^ii^	112.19 (18)
O8^iii^—Lu2—O8	89.08 (15)	O1—P2—O2^ii^	106.01 (18)
O8^vi^—Lu2—O8^vii^	89.08 (15)	O6^xi^—P2—O2^ii^	103.12 (18)
O8^iv^—Lu2—O8^vii^	90.92 (15)	O11—P3—O12	118.6 (3)
O8^iii^—Lu2—O8^vii^	180.0	O11—P3—O9^i^	105.0 (2)
O8—Lu2—O8^vii^	90.92 (15)	O12—P3—O9^i^	108.6 (3)
O8^vi^—Lu2—O8^viii^	89.08 (15)	O11—P3—O2	110.6 (2)
O8^iv^—Lu2—O8^viii^	90.92 (15)	O12—P3—O2	107.7 (2)
O8^iii^—Lu2—O8^viii^	90.92 (15)	O9^i^—P3—O2	105.49 (19)
O8—Lu2—O8^viii^	180.0	O8—P4—O4	116.6 (2)
O8^vii^—Lu2—O8^viii^	89.08 (15)	O8—P4—O10	107.9 (2)
O11^ix^—Lu3—O12	86.28 (18)	O4—P4—O10	113.3 (2)
O11^ix^—Lu3—O4	96.31 (13)	O8—P4—O6	108.8 (2)
O12—Lu3—O4	85.59 (14)	O4—P4—O6	110.62 (18)
O11^ix^—Lu3—O5	173.76 (15)	O10—P4—O6	97.91 (19)
O12—Lu3—O5	88.86 (16)		

Symmetry codes: (i) *x*−*y*+1/3, *x*−1/3, −*z*+2/3; (ii) *y*+1/3, −*x*+*y*+2/3, −*z*+2/3; (iii) −*x*+*y*+1, −*x*+1, *z*; (iv) −*y*+1, *x*−*y*, *z*; (v) −*x*+4/3, −*y*+2/3, −*z*+2/3; (vi) *y*+1/3, −*x*+*y*+2/3, −*z*−1/3; (vii) *x*−*y*+1/3, *x*−1/3, −*z*−1/3; (viii) −*x*+4/3, −*y*+2/3, −*z*−1/3; (ix) −*x*+*y*+1/3, −*x*+2/3, *z*−1/3; (x) −*x*+1, −*y*+1, −*z*; (xi) −*y*+2/3, *x*−*y*+1/3, *z*+1/3.
